# Advancing Workplace Civility: a systematic review and meta-analysis of definitions, measurements, and associated factors

**DOI:** 10.3389/fpsyg.2023.1277188

**Published:** 2023-11-09

**Authors:** Xue Peng

**Affiliations:** School of Marxism, Shandong Normal University, Jinan, China

**Keywords:** civility, Workplace Civility, systematic review, meta-analysis, relational, incivility

## Abstract

This research article focuses on the significance of Workplace Civility, defined as the respectful and courteous behavior exhibited by individuals toward their colleagues in the workplace. The primary objective of this study is to conduct a systematic review and a meta-analysis that synthesizes existing research by: (1) identifying operational definitions of the construct, (2) underlying the strongest correlations with other variables, (3) summarizing the effective strategies for promoting Workplace Civility, and (4) highlighting gaps in the literature, using the theory-characteristics-context-methodology (TCCM) framework. Multiple databases were meticulously searched, yielding 691 results, and ultimately 51 documents were included in the systematic review final sample following the application of predefined exclusion criteria. Then, a meta-analysis has been conducted including those studies with sufficient statistical data (*k* = 24) which allowed us to calculate 45 Effect Sizes. The review findings expose a notable dearth of research on Workplace Civility when compared to studies on incivility. This dearth highlights the pressing need for additional research endeavors to precisely define Workplace Civility, establish a robust theoretical framework, and develop reliable scales for its measurement. Related to the desirable correlates, organizational commitment, job satisfaction and mental health showed a high ES value, and for undesirable correlates, intention to quit showed a high ES value, while Emotional exhaustion only reached a medium ES value and physical symptoms showed a low ES value. Importantly, this study emphasizes that fostering civility in the workplace can yield significant benefits such as improved physical and mental well-being for workers, reduced burnout, and absenteeism rates. Thus, the promotion of civility in the workplace not only leads to healthier organizations but also enhances cost-efficiency, effectively averting the loss of both human and economic capital.

## Introduction

Workplace Civility refers to the respectful and courteous behavior exhibited by individuals toward their colleagues in the workplace (Clark and Walsh, 2016). It involves treating others with dignity, showing appreciation for their contributions, and refraining from any behavior that may be perceived as rude, aggressive, or disrespectful. Workplace Civility also encompasses active listening, empathy, and constructive communication, which contribute to fostering positive relationships, increasing job satisfaction, and improving organizational outcomes (Di Fabio and Kenny, 2018).

Civility refers to the practice of showing respect, courtesy, and politeness in our interactions with others. It’s about recognizing the dignity of others and adhering to social norms. In contrast, incivility is more than just a lack of civility; it’s the active display of behaviors that neglect or violate mutual respect, often leading to tension and conflict (Schilpzand et al., 2016). Rudeness, though related, is a subset of incivility. While rudeness pertains to overtly impolite actions, incivility can manifest in subtler ways, such as exclusion. Understanding the nuances between civility, incivility, and rudeness is crucial for fostering positive social interactions.

The theory of incivility can indeed be applied to understand civility, but with certain nuances in mind. Viewing incivility and civility through a dual spectrum approach can be beneficial. At the most basic level, understanding incivility can illuminate civility by contrasting it. The negative behaviors, attitudes, and disruptions detailed in the theory of incivility shed light on the nature of civility, helping to identify its presence through the absence or mitigation of these negative traits.

Moreover, delving deeper into the causes, manifestations, and impacts of incivility provides a richer comprehension of the environment and factors that promote civility. By discerning what drives incivility, stakeholders can strategize ways to encourage civility, leveraging a preventative rather than just reactive approach. For example, if a theory of incivility identifies isolation or lack of community engagement as a factor promoting incivility, efforts to foster inclusivity and community bonding can be seen as direct measures to promote civility.

However, a point of caution is essential. While the study of incivility can inform our understanding of civility, they are not mere opposites. Civility is not just the absence of incivility but has its own proactive attributes, characteristics, and dynamics. Using the theory of incivility as the sole lens to understand civility could risk oversimplifying or missing some of these unique nuances.

Despite its relevance, most empirical studies have primarily focused on incivility rather than exploring the positive and healthy characteristics of workplace environments (Gilin Oore et al., 2010; Guo et al., 2020). However, in recent years (Agarwal et al., 2023), there has been a shift toward analyzing and measuring Workplace Civility (Macintosh, 2002), moving away from exclusive focus on rudeness or incivility (Campbell et al., 2021).

Consequently, a systematic review of the literature on Workplace Civility is necessary to provide a comprehensive understanding of the topic and identify knowledge gaps. Workplace Civility plays a vital role in sustainable organizations by influencing employee well-being, job satisfaction, and organizational productivity. Nevertheless, despite the increasing interest in the topic, there remains a lack of consensus regarding its definition, measurement, and antecedents (Clark et al., 2013).

A systematic review can synthesize existing research, enable a critical evaluation of the evidence, which can inform the development of best practices and interventions. Furthermore, a meta-analysis can help identify strong relationship between Workplace Civility and sustainability outcomes, thereby informing targeted interventions and policies. By employing the theory-characteristics-context-methodology (TCCM) framework (Paul and Rosado-Serrano, 2019), pathways for future research can be suggested.

### Theoretical literature review

In the context of labor problems, the term “incivility” has gained widespread acceptance and usage (Cortina et al., 2001). Over the past two decades, scientific literature has extensively explored the issue of incivility among colleagues in the workplace (Agarwal et al., 2023). This topic has attracted the attention of both researchers and managers due to its high prevalence rate and the negative consequences it has on organizations and workers. These consequences include reduced performance, moral burnout, and estimated annual economic losses of approximately six billion dollars (Porath and Pearson, 2013; Nikstaitis and Simko, 2014; Schilpzand et al., 2016; Burke, 2018). However, there has been a shift in focus toward positive or healthy characteristics instead of solely focusing on negative or pathological aspects (Seligman and Csikszentmihalyi, 2000). As a result, in the last decade, many studies have sought to analyze and measure Workplace Civility rather than exclusively focusing on rudeness or incivility (Osatuke et al., 2009).

The concept of Workplace Civility has been widely discussed in the scientific literature (Belton and Dyrenforth, 2007). However, despite its importance, there is a significant lack of consensus regarding its definition and measurement. Some researchers view civility as the opposite of incivility (Hershcovis et al., 2017; Tsuno et al., 2017), while others allow individuals to interpret the term in their own way (Belton and Dyrenforth, 2007; Gilin Oore et al., 2010). Additionally, there is heterogeneity in how civility is measured, with some studies employing civility scales and others utilizing instruments to measure incivility. This lack of consensus makes it challenging to determine the exact meaning of civility in the workplace and the most effective way to measure it. Therefore, further research is needed to establish a clear and agreed-upon definition and measurement tool for this crucial concept. In the same vein, Workplace Civility plays a crucial role in promoting employee well-being and improving organizational outcomes. Hence, the most relevant relationships with personal and organizational work-related variables should be clarified, as well as the strategies for promoting civility at work.

Moreover, the Theory-Characteristics-Context-Methodology (TCCM) framework has emerged as a popular tool for researchers to identify gaps in specific areas of knowledge and provide clear directions for future research (Paul and Rosado-Serrano, 2019). This framework has been extensively used in literature reviews, and in the present study on Workplace Civility, the TCCM framework has been utilized to analyze the existing body of knowledge and suggest pathways for future research. The TCCM framework offers a structured approach to research by incorporating four key elements: theory, characteristics, context, and methodology. Researchers can use this framework to evaluate existing theories, identify unique characteristics of a particular phenomenon, examine contextual factors that may influence the phenomenon, and choose appropriate research methodologies to investigate it. Overall, the TCCM framework has proven to be an effective tool for advancing knowledge in specific fields and guiding future research (Singh and Dhir, 2019). The use of this framework in the present study would provide a comprehensive analysis of the existing literature on Workplace Civility and suggest several pathways for future research.

#### Research questions

The objective of the present study is to conduct a systematic review and a meta-analysis of the literature on Workplace Civility, combined with the TCCM framework, in order to answer the following research questions:

- What are the operational/analytical definitions of Workplace Civility?
- How has been Workplace Civility assessed?
- What are the correlates of Workplace Civility?
- What are the strong relationships between Workplace Civility and other work-related variables?
- What are the effective strategies and interventions for promoting and maintaining Workplace Civility?
- Using the TCCM framework, what are the gaps in the literature on Workplace Civility, and which areas require further research?

Answering these research questions will provide a comprehensive understanding of Workplace Civility, its antecedents, consequences, and effective strategies for promoting it. Additionally, identifying gaps in the literature and areas for future research will guide future investigations into this important topic.

## Method

### Search strategy

This systematic review aims to explore the concept of civility in the workplace by analyzing sources from a variety of disciplines, including the behavioral sciences, business studies, social sciences, psychology, and medicine. To accomplish this, a comprehensive search was conducted using multiple databases, including OpenGrey, PSICODOC, PsyARTICLES, Psychology and Behavioral Sciences Collection, PsycINFO, PubMed, PubPsych, Scopus, Google Scholar, and Web of Science (Core collection). As suggested, the PRISMA-ScR (Tricco et al., 2018) and the JBI Evidence Synthesis Handbook (Aromataris and Munn, 2020) have been followed.

**Figure 1:**
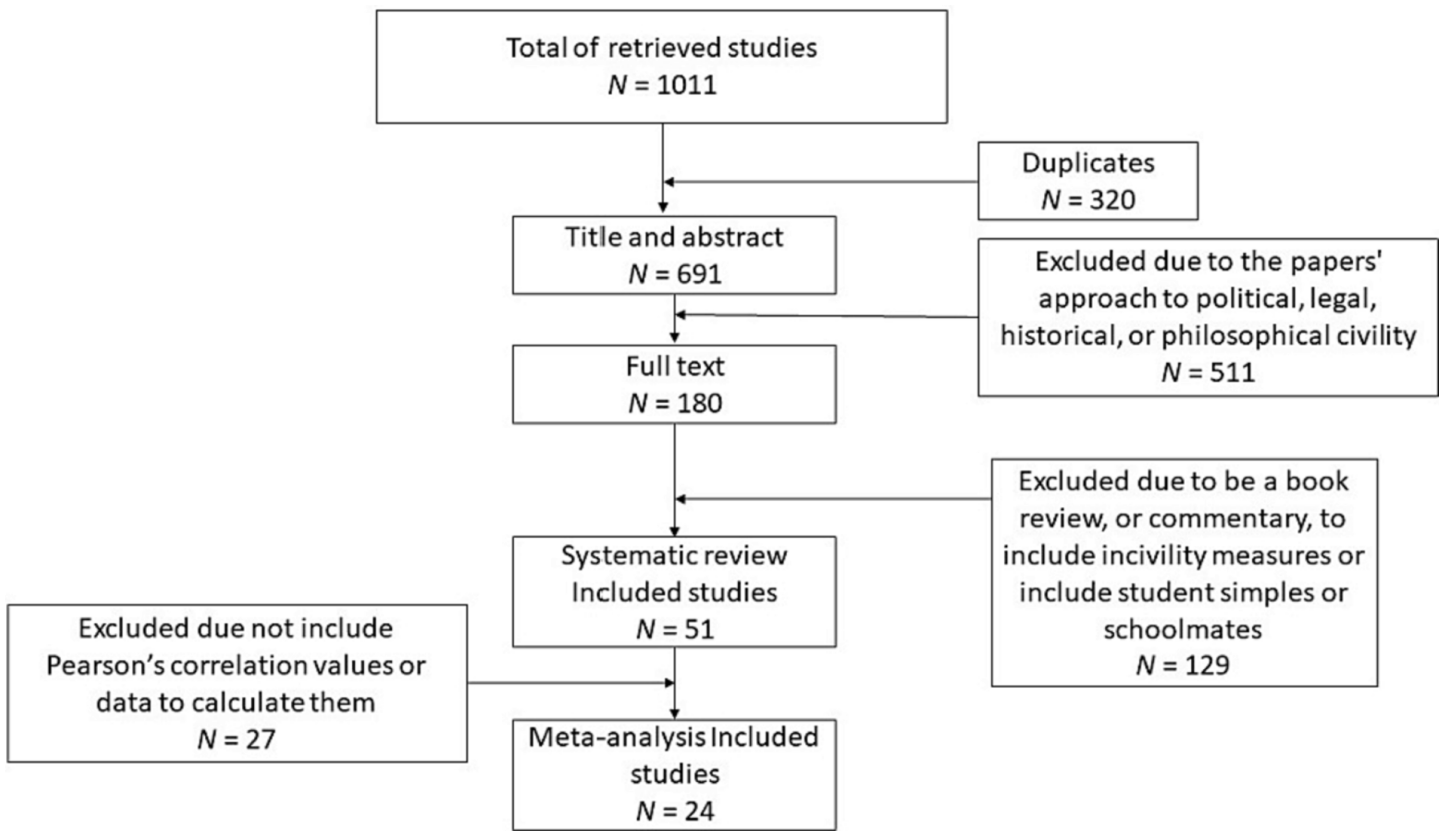
Flow diagram of the selection process.

A search protocol and strategy were developed for the term “Workplace Civility.” To ensure the least amount of bias throughout the search and to include the broadest variety of results, only the terms “civility” and “relation” have been used. All databases utilized the same search formula: (“relation*”) AND (“civility”). Different types of documents have been searched, as articles, doctoral theses, book chapters, conference papers, and other types of writing without any restrictions on the publication date, written in English, Portuguese, French, Italian, or Spanish. The last search was conducted in January 2023, yielding a total of 1,006 studies. Records from each database were imported into separate libraries within the EndNote 20.5 software, and these libraries were then merged into a single one, streamlining the removal of duplicates and the coding of studies. The selected articles either provided a definition, construct, or variable related to “civility” or included the concept to some extent. Works not written in English, as well as those focusing on politics and law, economic costs, students, and studies that solely measured incivility without any direct or indirect consideration of civility have been excluded. Figure 1 depicts the flow diagram of the selection process (refer to Figure 1). After removing duplicates (*N* = 315), the search returned 691 results.

### Inclusion and exclusion criteria

**Figure 2:**
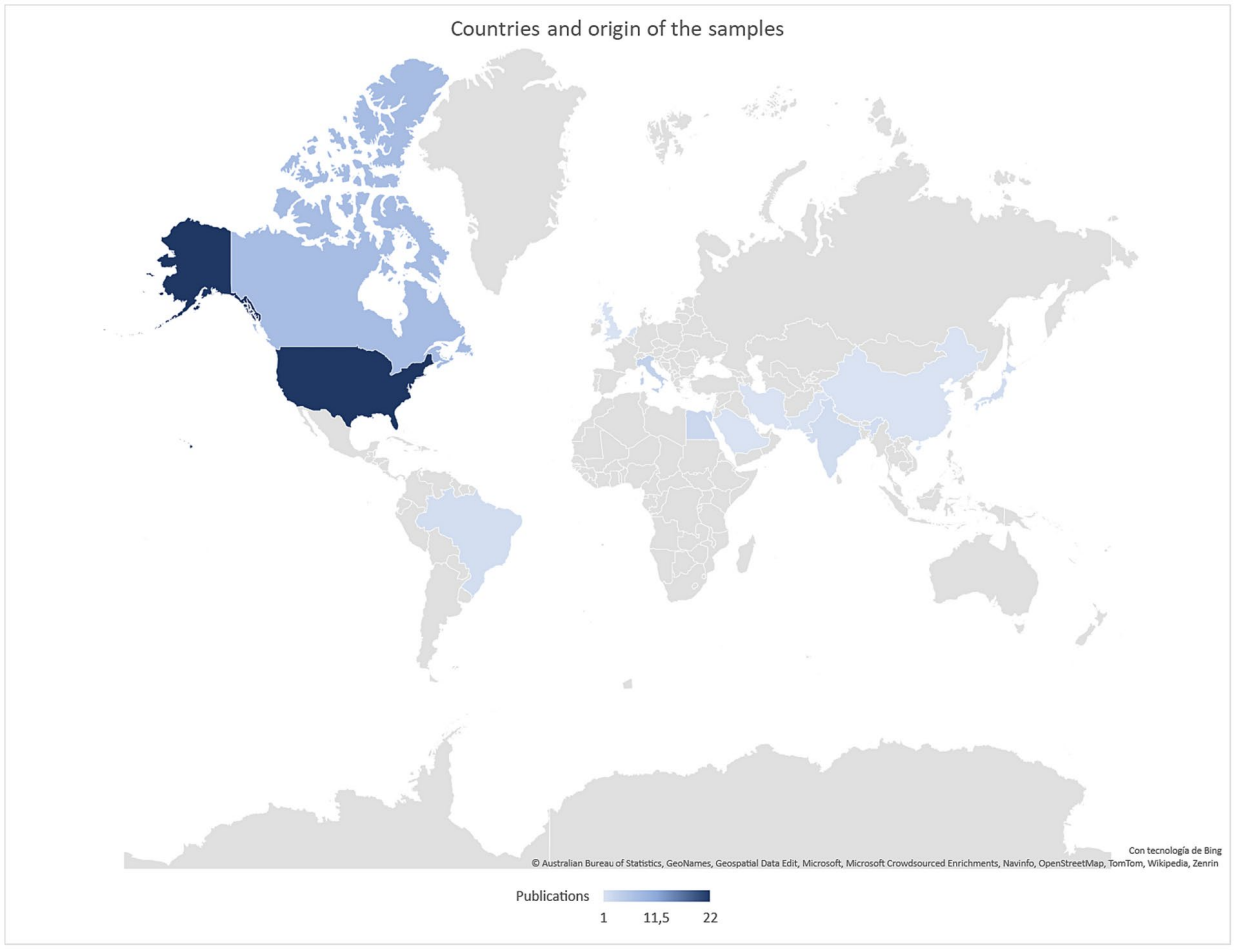
Country of origin of the samples.

The documents have been filtered in three stages according to the predefined inclusion and exclusion criteria mentioned above. In the first stage, the 691 results have been filtered based on title, abstract, keywords, and publisher, leading to the exclusion of 511 studies. The primary reasons for exclusion were the papers’ approach to political, legal, historical, or philosophical civility. Additionally, many papers emphasized rudeness rather than politeness. Subsequently, 180 documents that met the inclusion criteria or had characteristics that did not provide sufficient information to justify exclusion have been identified and retained. These documents were retrieved and examined in full text. The exclusion criteria have been applied (refer to Table 1), resulting in the exclusion of 129 studies and the selection of 51 documents for inclusion in the final sample (Figure 2).

**Table 1:** List of Inclusion and exclusion criteria.

Criteria	Inclusion	Exclusion
Type of document	Journal articles, books, book chapters, and doctoral theses	Editorials, book review, commentary, conferences, magazines, etc. Nonacademic documents. Unpublished studies.
Theoretical framework	Empirical (quantitative or qualitative) or theoretical study using behavioral definitions of the variables.	Documents focused on Workplace Civility from a legal, political, philosophical, sociological, historical, or anthropological sciences.
Concept	Workplace Civility	Civility at school, bullying or aggression. Civility in the social media or in the social networks. Documents focused on civility but only including *incivility* measures or analyses.
Participants	When empirical, workers as respondents.	Studies with samples of students or schoolmates, or community samples.
Language	Documents written in English, Portuguese, French, Italian, or Spanish.	Other languages.

### Coding and data extraction

To analyze the characteristics of the selected works, we developed a registration protocol based on the suggestions of Zeng et al. (2015). This protocol includes the following:

- External characteristics of research subjects and research methods used: author, year of publication, type of document, journal title, country of origin, source of funding, and conflict of interest disclosure.
- Methodological or procedural aspects that refer to how the research is conducted: type of research (qualitative, quantitative, mixed, theoretical, practical, or theoretical-practical), general framework (approach in relational civility or another theoretical framework), design, and methodological procedures.
- Basic characteristics of the three variables related to civility in the workplace that were studied: operational definition, inclusion in the study within a group of other variables, and method of measurement.

### Quality of systematic reviews

The methodological quality of the studies was evaluated. A quality assessment tool for quantitative studies (Sirriyeh et al., 2012) was used to further explore the works’ suitability. The following fields have been included in the data-base: (a) Explicit theoretical framework; (b) Statement of aims/objectives in main body of report; (c) Clear description of research setting; (d) Evidence of sample size considered in terms of analysis; (e) Representative sample of target group of a reasonable size; (f) Description of procedure for data collection; (g) Statistical assessment of reliability and validity of measurement tool (s); (i) Good justification for analytical method selected; (j) Strengths and limitations critically discussed. A Quality Global Rating for the papers was obtained ranging from (3) Strong, to (0) Weak.

### Meta-analytic procedures

In the selection of correlates for Workplace Civility in this meta-analysis, our choices were deeply anchored in the prevailing literature on organizational behavior. Job Satisfaction is frequently underscored as a primary outcome influenced by workplace civility; more civil environments can enhance satisfaction by fostering a sense of respect and worth (Spector, 1997). The inclusion of Emotional Exhaustion is predicated on findings suggesting that uncivil or disrespectful environments can contribute to employee burnout (Maslach et al., 2001). Organizational Commitment, a well-documented correlate, is influenced by the civility of the work environment, with increased civility promoting loyalty and attachment (Meyer and Allen, 1997). Similarly, Intention to Quit serves as a pivotal metric, representing direct consequences of incivility on staff retention (Mobley, 1977). Given the broad implications of workplace incivility on holistic well-being, Mental Health and Physical Illness or Symptoms were integrated based on evidence highlighting the association between workplace stressors and both mental and physical health outcomes (Karasek, 1990). In essence, these variables collectively furnish a comprehensive view into the multifaceted repercussions of civility, or its absence, within the workplace.

Hence, the following inclusion criteria have been fulfilled by those papers included in this meta-analysis: (a) to include relationships between Workplace Civility and its consequences; (c) to include a Pearson’s correlation coefficient or data that would allow us to calculate it. We obtained 24 empirical studies (from the total sample of 51 studies included in the previous systematic review), that allow us to and 45 independent effect sizes (ESs), which included 54 independent samples. Among the consequent variables, we coded Job satisfaction (k = 11 studies), Emotional Exhaustion (k = 10 studies), organizational commitment (k = 8 studies), intention to quit (k = 6 studies), mental health (k = 7 studies), and physical illness or symptoms (k = 3 studies). If a study includes more than one value of Workplace Civility-other variable correlation, only one of them has been included, guaranteeing the independence of ES (Moore, 2009; Di Fabio et al., 2016).

In this meta-analysis, the ES was Pearson’s correlation coefficient (r). ES values have been treated with Comprehensive Meta-analysis 2.0 (Borenstein et al., 2005) in order to be converted to Fisher’s *Z* transformation of *r*. The guideline to interpret the magnitude of ES was *r* < 0.20 = low ES value, r between 0.20 and 0.30 = medium ES value, and *r* > 0.30 = high (Hemphill, 2003). The 95% confidence interval was we also reported. Homogeneity analyses were carried out with *Q* statistics. Due to its shortcoming of poor power when a small number of studies are included, we provide *I*^2^. The *I*^2^index can be interpreted as the percentage of the total variability in a set of ESs due to true heterogeneity (Borenstein, 2022). Classic fail-safe N and Orwin’s fail-safe N have been used for estimations of publication bias.

## Results

Table 2 provides a comprehensive summary of the key features observed in the 51 studies that were included in the final selection for the Workplace Civility systematic review. The following subsections highlight some of the most important aspects revealed by these studies (Please, see Table 2).

**Table 2:** Included studies’ characteristics, operational definition and measurement of the construct, and variables included.

**Authors**	**Year**	**Doc.Type**	**Country**	**Sample size**	**Type of participants**	**Measurement of Workplace Civility**	**Operational definition of Workplace Civility**	**Variables included in the study**	**QGR**
1. Macintosh, G.	2002	A	CAN	220	University Employees	Three items *ad hoc*. *The travel counselor was courteous and polite*. *The travel counselor was rude.* (R) and *The travel counselor was easy to talk to.*	Meeting the customer’s standards of courtesy and accepted behavior.	Travel counselor dependability, expertise, familiarity, and civility, as well as clients’ trust; and clients’ satisfaction.	++
2. Porto and Tamayo	2003	A	BRA	1,110	Employees	*Scale of civility in organizations* (Porto and Tamayo, 2003): 41 items	Organizational Civil Behaviors includes 5 factors creative suggestions to the system, system protection, creation of a climate favorable to the organization in the external environment, self-training, and cooperation with colleagues.	Civility with a self-report survey of 64 items.	+++
3. Belton, L. W. and Dyrenforth, S. R.	2007	A	USA.	n.a.	Representatives of the Veterans Health Administration	Items from the All-Employee Survey (AES.).	Good behavior in the workplace,” including interpersonal respect, the fair resolution of disputes, and the tolerance and discrimination in the organization.	Civility as a part of the CREW: Civility, Respect, and Engagement in the workplace.	+
4. Ottinot, R. C.	2008	TH	USA	Study 1 (*N* = 189) Study 2 (*N* = 99).	Employees and coworkers	*Perceived Workplace Civility climate scale* 3 three dimensions including 16-item scale.	Three dimensions, (a) intolerance for incivility, (b) response, and (c) policies/procedures aimed at addressing incivility in the workplace	Primary participants (demographics, perceived Workplace Civility climate and all self-report variables). Coworkers (perceived Workplace Civility climate, interpersonal conflict at work, overall job satisfaction and the counterproductive work behaviors of the primary worker)	++
5. Osatuke, et al.	2009	A	USA	899	Employees of the Veterans Health Administration	*Civility survey* eight-item (Meterko et al., 2007).	Employee ratings of personal interest and respect from coworkers, cooperation or teamwork in the workgroup, fair conflict resolution, and valuing of individual differences by coworkers and supervisor.	Preintervention and postintervention changes in civility	+++
6. Moore, S. C.	2009	TH	USA	Four samples: 2006 *N* = 67,733; 2007 *N* = 70,592; and 2008 *N* = 69,290	Veteran’s Health Administration survey	*Civility survey* eight-item (Meterko et al., 2007).	Employee ratings of personal interest and respect from coworkers, cooperation or teamwork in the workgroup, fair conflict resolution, and valuing of individual differences by coworkers and supervisor.	Civility in Workplace and job satisfaction.	+++
7. Gilin Oore, D. et al.	2010	A	CAN	478	Healthcare employees from a 5-hospital system	Respect using a three-item scale (based on Siegrist et al., 2004).	To measure the positively valenced “civility,” respect from coworkers, supervisors and the organization.	Civility norms by individual measures of (1) coworker incivility, (2) supervisor incivility, and (3) workgroup respect; and by creating a dichotomous incivility measure: high incivility vs. Low incivility.	++
8. Ottinot, R. C.	2010	TH	USA	2,222	K-12 teachers	*Perceived Workplace Civility climate scale* 3 three dimensions including 16-item scale.	Three dimensions, (a) intolerance for incivility, (b) response, and (c) policies/procedures aimed at addressing incivility in the workplace	Workplace Civility Climate, at individual level and as a group-level construct, teacher experienced incivility, abuse, job satisfaction and affective commitment	+++
9. Leiter M. P. et al.	2011	A	CAN	1,173	Health care workers	*Civility survey* eight-item (Meterko et al., 2007).	Employee ratings of personal interest and respect from coworkers, cooperation or teamwork in the workgroup, fair conflict resolution, and valuing of individual differences by coworkers and supervisor.	Civility, burnout, job attitudes, management trust, and absences	+++
10. Leiter M. P. et al.	2012	A	CAN	1957	Health care providers	*Civility survey* eight-item (Meterko et al., 2007).	Employee ratings of personal interest and respect from coworkers, cooperation or teamwork in the workgroup, fair conflict resolution, and valuing of individual differences by coworkers and supervisor.	Civility, incivility, distress, and job attitudes	+++
11. Leiter M. P. et al.	2012	A	CAN	472	Nurses	*Civility survey* eight-item (Meterko et al., 2007).	Employee ratings of personal interest and respect from coworkers, cooperation or teamwork in the workgroup, fair conflict resolution, and valuing of individual differences by coworkers and supervisor.	Civility, incivility, burnout, work engagement and coworkers’ and supervisors’ support.	++
12. Walsh et al.	2012	A	USA	2,711	Employees	*Civility norms questionnaire-brief* (Walsh et al., 2012). 4-items Likert-type scale	Civility is considered as the individual perceptions of civility norms, or the degree to which norms for respectful treatment exist	Civility, incivility, organizational justice, job satisfaction, commitment, and intentions to quit.	+++
13. Clark, C.	2014	A	USA	Time 1 (*n* = 54), Time 2 (*n* = 68), and Time 3 (*n* = 66).	Nursing students	*Nursing civility scale*: Four quantitative items measured nursing students’ perceptions.	Nursing students’ perceptions includes (1) level of civility in the nursing program; (2) quality of student-faculty relationships; (3) quality of student–student relationships; and (4) number of hours spent per week in stress-reducing activities.	Stress, coping, faculty-student, and student–student relationships, and ways to promote civility in nursing education	+++
14. Mcgonagle, A. K. et al.	2014	A	USA	Sample 1 (*N* = 421) Sample 2, (*N* = 964)	Mechanical workers and non-management employees	*Civility norms questionnaire-brief* (Walsh et al., 2012). 4-items Likert-type scale	Individual perceptions of civility norms, or the degree to which norms for respectful treatment exist	Civility norms indirectly related to safety outcomes (i.e., unsafe behaviors and on-the-job injuries) through associations with specific psychosocial safety climate dimensions (i.e., management safety climate, coworker safety climate) and work safety tension (felt conflict between job tasks and safety)	+++
15. Montalvo, L.	2014	TH	USA	*K* = 17	Articles on civility	n.a.	n.a.	Civility, nurses’ well-being and organizational commitment, nurses’ productivity, patients’ perception of treatment and health care outcomes.	n.a.
16. Leiter M. P. et al.	2015	A	CAN	FLMs (*N* = 157) staff (*N* = 1,624).	First-line managers and frontline staff of healthcare organizations.	*Civility survey* eight-item (Meterko et al., 2007).	Employee ratings of personal interest and respect from coworkers, cooperation or teamwork in the workgroup, fair conflict resolution, and valuing of individual differences by coworkers and supervisor.	Attachment anxiety and avoidance, professional efficacy, trust, psychological safety, civility; incivility, exhaustion, and cynicism. Workgroup civility as independent variable.	+++
17. Hernandez, W., et al.	2015	A	USA	3,674	Representatives of the Veterans Health Administration	*Civility survey* eight-item (Meterko et al., 2007).	Employee ratings of personal interest and respect from coworkers, cooperation or teamwork in the workgroup, fair conflict resolution, and valuing of individual differences by coworkers and supervisor.	Managerial self-awareness, supervisor burnout, supervised workgroup climate, Workplace Civility and Psychological Safety.	+++
18. Porath, C. L. et al.	2015	A	USA	Study 1, Time 1, (*N* = 46). Time 2 (*N* = 42). Study 2, *N* = 181	Biotechnology firm Employees and students	One Likert question: “To what extent is this person civil?	Interactions, such as feeling listened to, receiving acknowledgment, credit, or thanks, and being asked questions humbly, should ignite positive feelings, such as pride, esteem, or dignity.	Perceptions of Leaders as (a) warm and (b) Competent, and Workplace Civility as independent variable.	+++
19. Clark, O. L., and Walsh, B. M.	2016	A	USA	239	University employed students	*Civility norms questionnaire-brief* (Walsh et al., 2012). 4-items Likert-type scale	Individual perceptions of civility norms, or the degree to which norms for respectful treatment exist	Organizational constraints, interpersonal Deviance, and Team civility climate.	++
20. Di Fabio, A. et al.	2016	A	ITA	261	Employees from public and private organizations	*Workplace relational civility scale* a self-report mirror instrument of 26 Items that assesses Relational Civility at work.	Three interrelated components: relational readiness, relational culture, and relational decency	Three dimensions relational decency; relational culture; and relational readiness. Acceptance of change, well-being (hedonic well-being as well as eudemonic well-being), and personality traits.	++
21. Di Fabio, A., and Gori, A.	2016	A	ITA	115	Employees from public and private organizations	*Workplace relational civility scale* a self-report mirror instrument of 26 Items that assesses Relational Civility at work.	Behaviors like treatin Three interrelated components: relational readiness, relational culture, and relational decency	Workplace Relational Civility, Organizational citizenships behavior, Prosocial organizational Behavior, Intrapreneurial Self-Capital, Flourishing, Satisfaction with life, Self-esteem, Perceived social support, Trait emotional intelligence, and Workplace incivility.	+
22. Gazica, M. W. and Spector, P. E.	2016	A	USA	386	Employees	*Perceived Workplace Civility climate scale* 3 three dimensions including 16-item scale.	Three dimensions, (a) intolerance for incivility, (b) response, and (c) policies/procedures aimed at addressing incivility in the workplace	Workplace Civility climate, safety climate, and violence prevention climate. Accidents, musculoskeletal disorders, physical and nonphysical violence, incivility exposure, and interpersonal conflict.	++
23. Laschinger H. K. S. and Read, E. A.	2016	A	CAN	993	New graduate nurses	*Civility norms questionnaire-brief* (Walsh et al., 2012). 4-items Likert-type scale	Individual perceptions of civility norms, or the degree to which norms for respectful treatment exist	Civility norms as an independent variable. Authentic leadership, person-job fit, coworker incivility and subsequent emotional exhaustion.	+++
24. Alamelu, R. et al.	2017	A	IND	200	Banking Employees	Questionnaire *ad hoc*, without information about the length, type of items or its psychometric properties.	Norms and rules to be adhered when dealing with others.	Workforce Civility as a construct integrated by five factors: overall workforce civility, effective work etiquette, cost and reward, communication, and conflict and resolution.	++
25. Costa, V. F. et al.	2017	A	BRA	302	Employees of a manufacturer of home appliances in Rio Grande do Sul	*Scale of civility in organizations* (Porto and Tamayo, 2003): 41 items	Five factors: creative suggestions to the system, system protection, creation of a climate favorable to the organization in the external environment, self-training, and cooperation with colleagues.	Organizational Citizenship Behavior as a construct integrated by 5 factors, one of them is civility. Organizational Values, job satisfaction.	++
26. Doucette, W. C. and Tolley, R. L.	2017	BCH	USA	n. a.	n. a.	Opinion Survey on Civility in the Workplace	Consistent mindful speech and the first step toward higher levels of empathy and increased cooperation in the workplace	Civility as a construct characterized by personal dignity and respect.	+
27. Gillen, P. A. et al.	2017	A	IRE-SWE-ENG.	4,116	Participants of five studies included in the meta-analysis	n.a.	n.a.	Experiences of civility as the inverse of incivility and as an indirect measure of bullying victimization.	++
28. Tsuno, K. et al.	2017	A	JAP-CAN	Sample 1 (*N* = 2,191) and Sample 2 (*N* = 1,071)	Japanese employees and Canadian health care employees	*Civility survey* eight-item (Meterko et al., 2007). The 8-item Civility Scale Japanese version measures the perceptions of civility within a workgroup and across an organization.	Employee ratings of personal interest and respect from coworkers, cooperation or teamwork in the workgroup, fair conflict resolution, and valuing of individual differences by coworkers and supervisor.	Workgroup Civility, and demographic characteristics.	+++
29. Yanchus et al.	2017	A	USA	10,997	Mental health employees	*Civility subscale*, average of the subscale that contained four items.	Not defined. Instead, it is considered that Civility includes respect; conflict resolution; cooperation, and diversity acceptance.	Civility (courteous and respectful workplace behaviors) and supervisory support. Job satisfaction, emotional exhaustion, turnover intention, and turnover plans.	+++
30. Hostetler, T. J.	2017	TH	USA	4,037 and 1,264	Registered nurses and employees of two healthcare organizations	Overall civility rating (Section 9) from the *Organizational civility scale* (Clark et al., 2013). 108-item scale	Antonym of incivility. There is not an explicit definition.	Nurse perceptions of civility resources, incivility, stress, coping, and job satisfaction	+++
31. Hutchinson, D. M. et al.	2018	A	USA	420	Employees	*Perceived Workplace Civility climate scale* 3 three dimensions including 16-item scale.	Three dimensions, (a) intolerance for incivility, (b) response, and (c) policies/procedures aimed at addressing incivility in the workplace	Civility climate as a second order factor of the general safety climate. Interpersonal conflict, workplace aggression, exposure to uncivil behavior, workplace accidents, and job satisfaction.	++
32. Nagy, M.	2018	BCH	USA	n.a.	n. a.	n.a.	n.a.	Theoretically developed the notion of civility, and its benefits and costs compared to diversity training.	
33. Palazzeschi, L.	2018	A	ITA	204	Employees of care organizations	*Workplace relational civility scale* a self-report mirror instrument of 26 Items that assesses Relational Civility at work.	Three interrelated components: relational readiness, relational culture, and relational decency	Workplace Relational Civility and its three dimensions relational decency; relational culture; and relational readiness as a dependent variable.	++
34. Clark, C., Sattler, V., and Barbosa-Leiker, C.	2018	A	USA-CAN	393	Attendees from one international nursing conference	*Workplace Civility index* (Clark, et al., 2018) is a 20-item, Likert-type survey consisting of 20 elements. Respondents assess the perceived frequency of civil workplace interactions.	Not defined, but the construct is suggested as opposite to Incivility.	Self-reflection.	++
35. Abd-Elrhaman, E. S. A., and Ghoneimy, A. G. H.	2019	A	EGY	176	Nurses in critical care units	*Workplace Civility index* (Clark, et al., 2018) is a 20-item, Likert-type survey consisting of 20 elements. Respondents assess the perceived frequency of civil workplace interactions.	Not defined, but the construct is suggested as opposite to Incivility.	The knowledge and practices regarding professional nursing ethics, and level of Workplace Civility before and after the implementation of professional nursing ethics program	+
36. Clark, C.	2020	CP	USA-CAN	393	Nursing faculty and practice-based nurses	*Workplace Civility index* (Clark, et al., 2018) is a 20-item, Likert-type survey consisting of 20 elements. Respondents assess the perceived frequency of civil workplace interactions.	Not defined, but the construct is suggested as opposite to Incivility.	The perceived frequency of civil workplace interactions	++
37. Liu et al.	2020	A	USA-ISR	Study 1: (*N* = 432) Study 2: (*N* = 377)	Management undergraduate students and surgical teams from a large tertiary health care center in Israel.	*Team civil communication*: number of text communications that included elements of courteousness, graciousness, consideration, support and/or encouragement. *Team civil communication*: idem expressed by team members from the preparation stage to the end of the surgical operation.	Workplace Civility includes verbal civility (e.g., civil communication) and non-verbal civility (e.g., civility conveyed from facial expressions and body gestures).	Verbal civility at work operationalized as work-based civil communication, such as interpersonal communication characterized by courteousness, graciousness, consideration, support and/or encouragement in work-related contexts	++
38. Gori and Topino	2020	A	ITA	130	Employees of public and private organizations	*Workplace relational civility scale* a self-report mirror instrument of 26 Items that assesses Relational Civility at work.	Three interrelated components: relational readiness, relational culture, and relational decency	Psychological factors [predisposition to change, workplace relational civility (others with me) and job satisfaction].	+
39. Oppel, E. M., and Mohr, D. C.	2020	A	USA	Nurses (*N* = 6,019) Patients (*N* = 38,619)	Nursing of the Veterans Health Administration, and Patients	*Providers’ civility climate* eight-item scale (Leiter et al., 2011), participants’ perceptions of civility within their workgroup and their workplace as a whole. *Civility toward patients*: Agreement to items asking how often nurses treated patients with courtesy and respect.	Civility climate includes: (a) respect and acceptance, (b) cooperation, (c) supportive relationships between coworkers, and (d) fair conflict resolution.	Providers’ civility climate, overall hospital rating, patients’ intent to recommend, patients’ willingness to return and civility toward patients.	+++
40. Erum, H., Abid, G., Contreras, F., and Islam, T.	2020	A	n. a.	335	Employees	*Civility scale* Porath and Erez’s (2007) four-item scale. Sample items include “Do your co-worker treat you with respect?” and “Do your co-worker treat you with dignity?”	Employees’ courteous, respectful, and caring behavior toward each other in formal and informal social relations.	Family motivation, civility, affective commitment, organizational citizenship behavior and self-efficacy.	++
41. Der Kinderen, S., Valk, A., Khapova, S. N., and Tims, M.	2020	A	NLD	312	Mental health care employees	*Civility norms questionnaire-brief* (Walsh et al., 2012). 4-items Likert-type scale	Individual perceptions of civility norms, or the degree to which norms for respectful treatment exist	Servant leadership, eudemonic well-being, and Workplace Civility climate.	++
42. Liu, L.	2020	TH	CHN	723.	Micro, small, and medium enterprises Employees	*Workplace relational civility scale* a self-report mirror instrument of 26 Items that assesses Relational Civility at work.	Three interrelated components: relational readiness, relational culture, and relational decency	Health-promoting leadership, employee health, Workplace Civility, and workplace ostracism on employee engagement, and employability.	+++
43. Campbell, et al.	2021	A	USA	1,043	Staff and faculty in medical, nursing, pharmacy, and health professions schools	*Organizational civility scale* (OCS) consisting in 88-item, which measures the continuum of professional and unprofessional behaviors experienced by employees.	Frequency of incivility, overall civility rating, perceptions of organizational climate, importance of civility resources, and existence of civility resources.	Organizational civility and other variables, such as feelings about current employment, employee satisfaction, sources of stress, coping strategies, and overall levels of stress and overall coping ability.	+++
44. Sawada, et al.	2021	A	JAP	Sample 1 (*n* = 17–22), Sample 2 (*n* = 9–13), Sample 3 (*n* = 6–10)	Nurses, medical doctors, and other psychiatric professionals	*Civility survey* eight-item (Meterko et al., 2007).	Employee ratings of personal interest and respect from coworkers, cooperation or teamwork in the workgroup, fair conflict resolution, and valuing of individual differences by coworkers and supervisor.	Social climate, civility scale, and work engagement (UWES) have been assessed over time	++
45. Alam, M., Fozia, G. U. L., and Imran, M.	2021	A	PAK	340	Employees manufacturing sector	*Civility*: four-item scale assessing civility was used. Example item was “Do your co-workers treat you in a polite manner?	Prescribed interpersonal actions that verified value and be in love with others to create useful affairs at workplace.	Ethical leadership, civility, work engagement and organizational commitment.	++
46. Gupta, A. and Singh, P.	2021	A	IND	363	Employees technology companies	*Civility survey* eight-item (Meterko et al., 2007).	Employee ratings of personal interest and respect from coworkers, cooperation or teamwork in the workgroup, fair conflict resolution, and valuing of individual differences by coworkers and supervisor.	Job crafting, Workplace Civility, work engagement and change perception, general life satisfaction and intention to quit, considered as outcomes.	++
47. Hossny, E. K., and Sabra, H. E.	2021	A	SAU	139	Nurses	*Perceived Workplace Civility climate scale* 3 three dimensions including 16-item scale.	Three dimensions, (a) intolerance for incivility, (b) response, and (c) policies/procedures aimed at addressing incivility in the workplace	Nurses’ perception to Workplace Civility climate on nurse–physician collaboration	+
48. Ahmed Elsayed, W. et al.	2021	A	EGY	150	Nurses	*Perceived Workplace Civility climate scale* 3 three dimensions including 16-item scale.	Three dimensions, (a) intolerance for incivility, (b) response, and (c) policies/procedures aimed at addressing incivility in the workplace	Leadership competencies, Workplace Civility climate, and mental wellbeing scale	+
49. Savadkouhi, S., Oreyzi, H., and Asgari, K.	2021	A	IRN	75	Gas company employees	*Relational energy scale* Owens et al.’s (2016), five items.	Civility at work is identified as an antecedent of Employees’ Relational energy.	CREW intervention and relational energy.	+
50. Ahmed, E. A. A.	2022	A	EGY	127	Head nurses and staff nurses	*Perceived Workplace Civility climate scale* 3 three dimensions including 16-item scale.	Three dimensions, (a) intolerance for incivility, (b) response, and (c) policies/procedures aimed at addressing incivility in the workplace	Civility knowledge, Workplace Civility climate, nursing professional value, and legal and ethical issue knowledge	++
51. Apaydin, et al.	2022	A	USA	3,216	Primary care provider Employees of the Veterans Health Administration	*Perceived Workplace Civility* five items asking about cooperation, accepting differences, conflict resolution, and psychological safety.	Civility includes courtesy, politeness, and respect.	Outcomes: burnout; predictors: Workplace Civility and gender; controls: race, ethnicity, VA tenure, and supervisory status.	+++

CREW, civility, respect, and engagement in the workplace; HELP, high entrepreneurship, leadership, and professionalism; VHA, Veteran’s Health Administration; WRC, workplace relational civility, n. a., not available. Document Type: A, Article; BCH, book chapter; CP, conference paper; TH, thesis. Country: BRA, Brazil; CAN, Canada; CHN, China; EGY, Egypt; ENG, England; IND, India; IRE, Ireland; IRN, Iran; ISR, Israel; ITA, Italy; JAP, Japan; NLD, Netherlands; PAK, Pakistan; SAU, Saudi Arabia; SWE, Sweden; and USA, United States. QGR, Quality Global Rating for this paper. +++, strong, ++, moderate, +, weak.

### Description of primary studies

The majority of research in this field has been conducted across diverse regions, including North America (*n* = 28), Europe (*n* = 6), while less quantity of studies conducted in Asia (*n* = 5), and South America (*n* = 1) (refer to Figure 1). The studies spanned a time period from 2002 to 2022, with a total of 19 studies published within the past 5 years, indicating a growing interest in the topic. The primary mode of publication studies was scientific journal articles (*n* = 42), although a few were presented as book chapters or doctoral theses. It is worth mentioning that one of the included studies constituted a brief meta-analysis of five studies (Gillen et al., 2017), and other constitutes a systematic review of interventions (Montalvo, 2014), adding further value to the body of knowledge in this area. Overall, these findings shed light on the multifaceted nature of Workplace Civility and highlight the geographical distribution, temporal trends, and publication characteristics of the selected studies. The subsequent sections will delve deeper into the specific findings and insights derived from the systematic review.

The methodology employed in the studies that contributed to the theoretical framework of this research varied, with the majority being empirical research (*n* = 48). A significant portion of the works combined theoretical and empirical approaches, while the remaining studies (*n* = 3) were purely theoretical. Quantitative methodology was more frequently utilized than qualitative methodology, indicating a preference for data-driven analysis.

Cross-sectional designs were prevalent as research design (*n* = 46), providing a snapshot of Workplace Civility at a specific point in time. Convenience sampling or snowball sampling techniques were the prevalent methods for participant selection in these studies. Additionally, several studies were associated with the Veteran Health Administration’s All-Employees Survey in the United States, featuring larger samples or multi-samples’ datasets that encompassed data on the Civility, Respect, and Engagement in the Workforce training program.

Quality assessment of empirical studies showed that only 22 pieces of research reached the strongest values using the tool for quantitative studies (Sirriyeh et al., 2012). Reduced samples, with only a cross-sectional design, convenience sampling procedures and lack of adequate justification of the participants inclusion and the analytical procedures were the main reasons for medium and lower values of Quality Global Rating, as displayed in Table 2.

#### What are the operational/analytical definitions of Workplace Civility?

A noteworthy finding from our analysis is the variation in definitions of Workplace Civility across the included studies. As can be seen in Table 2, while many articles did not explicitly define workplace politeness and focused on describing impoliteness as the opposite of politeness, a closer examination of the selected studies uncovered distinct conceptualizations of civility within the workplace. These definitions encompassed various levels, including:

- Interpersonal Level: Some studies emphasized individual perceptions of civility, respect, and commitment in the workplace (e.g., CREW - Civility, Respect, and Commitment in the Workplace). Others explored cooperation and respect among workers (e.g., Civility in the Workplace and Workplace Relational Civility).
- Group or Organizational Level: Several studies examined shared perceptions of civility within a group or organization (e.g., Team Civility Climate). Additionally, there were investigations into rules of civilized behavior (e.g., Civility Norms), shared perceptions of the civility environment (including political practices and formal and informal rules such as Safety Climate), and pro-social behaviors that promote the proper functioning of the company (e.g., Organizational Civil Behaviors).

The operational definition of Workplace Civility often coincides among the different studies, showing the most relevant facets as respect, courtesy, and tolerance.

#### How has been Workplace Civility assessed?

The studies included in this review utilized various methods to measure Workplace Civility, as can be seen in Table 3. These methods can be grouped into several categories.

**Table 3:** Mean weighted effect sizes for meta-analysis.

Variables	*k*	Fisher’s *Z*	SD	95% CI		*Q* (df)	*I*^2^	Publication bias	
				Ll	Ul			Classic fail-safe *N*	Orwin’s fail-safe *N*
Desirable correlates									
Job satisfaction	11	0.584***	0.007	0.571	0.598	358.154 (10) ***	97.2	1,632	196
Organizational commitment	8	0.601***	0.013	0.576	0.626	365.8 (7) ***	98.06	3,496	147
Mental health	7	0.420***	0.016	0.388	0.452	406.44 (6) ***	98.52	1,045	87
Undesirable correlates									
Physical symptoms	3	−0.039***	0.022	−0.083	0.005	108.13 (108) ***	98.15	3	6
Emotional exhaustion	10	−0.229***	0.007	−0.243	−0.215	63.16 (9) ***	85.75	2026	209
Intention to quit	6	−0.361***	0.008	−0.377	−0.346	30.96 (5) ***	83.85	1942	205

k, number of correlations. 95% CI, 95% confidence interval around Weighted r; Ll, lower limit; Ul, upper limit. Q (df), chi square test for homogeneity (degrees of freedom); I^2^, percentage of variance beyond the sampling error. *p < 0.05. **p < 0.01. ***p < 0.001.

##### Standardized questionnaires or scales

Some studies employed questionnaires or scales specifically designed to assess Workplace Civility. These instruments included measures such as the Civility Norms Questionnaire-Brief, Scale of Civility in Organizations, CREW Civility Scale, Workplace Relational Civility Scale, Organizational Citizenship Behavior Checklist, and others. These tools provided structured assessments of civility levels in the workplace. The most common procedure to assess Workplace Civility are the standardized questionnaires.

##### Behavioral observations

In certain studies, researchers observed behavior to capture Workplace Civility. For example, the Team Civil Communication approach involved counting courteous text communications during teamwork. This method provided direct observations of civil behavior in real-life work settings.

##### Extracted subscales or items

Some studies extracted subscales or individual items related to civility from broader questionnaires. For instance, researchers utilized a scale derived from the All-Employee Survey or included specific items related to “civility” from the General Safety Scale. This approach allowed for a targeted assessment of civility within the context of larger surveys.

##### Assessment of incivility

In a few cases, studies focused on assessing workplace incivility rather than civility itself. These investigations utilized questionnaires or scales explicitly designed to measure incivility, such as the Workplace Incivility Scale. While not directly assessing civility, these measures provided insights into the presence and impact of uncivil behavior in the workplace.

##### Theoretical studies

Finally, some theoretical studies included in this review did not utilize specific measurement instruments. Instead, they focused on theoretical discussions and did not involve empirical data collection.

By employing a range of measurement approaches, the reviewed studies enhanced our understanding of Workplace Civility. Future research should aim to provide more detailed descriptions of the measurement instruments used, facilitating transparency, and enabling comparisons across studies. The subsequent sections will explore the specific findings derived from these diverse measurement methods and their implications.

#### What are the correlates of Workplace Civility?

Workplace Civility was examined in the included studies in two primary ways: as a predictive variable for desired or undesired outcomes, and as a component of intervention programs aimed at promoting civil behavior among employees (Please, see Table 2).

When studied as a predictive variable, Workplace Civility was frequently found to correlate with various important outcomes such as job satisfaction, work engagement, burnout, abuse, affective commitment, turnover intentions and plans, and absences, among others. These findings suggest that the level of civility in the workplace can significantly impact employees’ experiences and well-being. The meta-analytic results provided a more objective picture of the relationships between Workplace Civility and work-related variables.

Moreover, Workplace Civility was investigated as part of intervention programs, particularly the Civility, Respect, and Engagement in the Workforce (CREW) proposal. In this context, researchers assessed civility as a variable to examine changes that occurred between pre-and post-intervention phases. This approach provided valuable insights into the effectiveness of interventions in promoting and fostering a more civil work environment. The examination of Workplace Civility, in both predictive and intervention contexts, offers a comprehensive understanding of its implications and highlights the potential benefits of cultivating a civil work culture. The subsequent sections will delve further into the specific findings and implications derived from these relationships.

#### What are the strong relationships between Workplace Civility and other work-related variables?

The meta-analytic results shed light on the relationships between Workplace Civility and various work-related factors in a more systematic and objective manner. Referring to Table 3, a comprehensive analysis was performed on data from 24 studies, which enabled us to examine a broad range of correlates associated with Workplace Civility. This includes factors like job satisfaction, emotional exhaustion, organizational commitment, intention to quit, mental health, and physical symptoms. Focusing on the correlates that are generally seen as desirable in a workplace, organizational commitment, job satisfaction, and mental health stood out with a high Effect Size (ES) value. According to Hemphill’s guidelines (2003), these values ranged between 0.601 and 0.420, and all were statistically significant, reinforcing their robustness. On the flip side, when examining the correlates deemed less desirable in association with Workplace Civility, different patterns emerged. Specifically, ‘intention to quit’ presented a high ES value, suggesting a strong inverse relationship with civility. Meanwhile, ‘emotional exhaustion’ had only a medium ES value, and ‘physical symptoms’ exhibited a low ES value. This indicates varying degrees of negative correlation with Workplace Civility. Notably, all ES values aligned with anticipated outcomes: the positive or desirable correlates had positive ES values, suggesting a direct relationship with Workplace Civility, while the negative or undesirable ones had negative ES values, implying an inverse relationship. This consistency reinforces the reliability of our findings and highlights the essential role of civility in shaping workplace dynamics. Finally, Table 3 shows the results for the *Q* statistics analyses and *I*^2^. Both values revealed significant heterogeneity across studies, with percentages of explained variance due to this heterogeneity ranging from 836% for the Workplace Civility-Intention to quit relationship to 99.6% for Workplace Civility-Mental health. These results suggested that a high percentage of the variability among the studies is due to true heterogeneity, claiming for potential moderator variables analyses. These analyses cannot be carried out due to a reduced number of studies in each meta-analysis. Finally, the publication bias findings showed a large number of studies required in each category, except for the meta-analysis on Physical Symptoms, indicating that this finding should be taken with caution.

## Discussion

Theoretical frameworks such as positive psychology and the Positive Psychology have emphasized the significance of Workplace Civility in promoting employees’ well-being and achieving desired group and organizational outcomes, including performance and productivity (Ahmed, 2022). Consequently, recent research has increasingly focused on investigating the impact of Workplace Civility, with particular interest in the USA and Canada where 75% of the publications originate. This systematic literature review aims to address the research questions:

### What are the operational definitions of Workplace Civility?

Workplace Civility has emerged as a multifaceted concept in recent research, encompassing various behaviors and perceptions across both individual and organizational levels. At the individual level, definitions such as Civility, Respect, and Commitment in the Workplace (CREW) highlight the importance of positive interactions between colleagues. This emphasizes courtesy, teamwork, and mutual respect (Gilin Oore et al., 2010; Leiter M. P. et al., 2011; Laschinger et al., 2012; Leiter et al., 2012). Another perspective underscores the importance of cooperation and mutual respect, emphasizing the interconnected nature of Workplace Civility (Yang et al., 2014; Hernandez et al., 2015).

On the organizational front, shared perceptions play a pivotal role. Team Civility Climate, shaped by group norms and values, reflects the collective influence on individual behaviors (Clark and Walsh, 2016). Civility Norms, both written and unwritten, guide behavior toward harmony and respect (McGonagle et al., 2014). Additionally, aspects like Safety Climate, which focuses on shared safety perceptions, further impact workplace behavior (Hutchinson et al., 2018). Finally, Organizational Civil Behaviors (Hostetler, 2017; Campbell et al., 2021), represent actions that benefit the organization as a whole, such as helping peers or volunteering.

In essence, Workplace Civility is a blend of individual attitudes, group perceptions, and overall organizational behaviors. Understanding this dynamic is crucial for creating a cohesive and productive work environment. Future studies should further examine these components and their influence on key organizational metrics.

### How has been Workplace Civility assessed?

In organizational research, accurately measuring abstract concepts like Workplace Civility is crucial for reliable findings. The present review emphasizes the value of using standardized questionnaires to study this construct. These instruments, often rigorously validated, offer a clear and consistent understanding of Workplace Civility (Meterko et al., 2007; Osatuke et al., 2009; Ottinot, 2010; Di Fabio and Gori, 2016). Their consistent use ensures researchers are on the same page when discussing civility, allowing for easier comparison across different studies. Using a standardized set of questions rooted in theory and prior research enhances the quality of insights (Tsuno et al., 2017; Sawada et al., 2021). In summary, anchoring research on Workplace Civility with standardized tools is more than a best practice. It is an assurance of clarity, consistency, and meaningful contributions to the ongoing dialogue in organizational studies. The insights from our review reiterate the significance of this approach and the criticality of its widespread adoption.

### What factors are associated with Workplace Civility?

The present review of Workplace Civility highlights its intricate connections to both personal traits and the broader work environment. Certain individual characteristics, such as gender, humor, and collectivism, emerge as key factors influencing civility. Interestingly, the longer someone stays in an organization, the more civil they tend to become, potentially due to deeper emotional bonds formed over time.

Workplace Civility’s effects are wide-ranging. On the positive side, when there’s more civility in a workplace, employees often feel more job satisfaction, are happier with their pay, and engage more in helpful behaviors. They also feel more satisfied with life in general. On the flip side, civility also lessens negative outcomes. For instance, in a more civil workplace, employees feel less burnt out and stressed, as noted (Day and Leiter, 2018). Gilin Oore et al. (2010) also found that civility can act as a protective shield against job pressures.

Looking at the bigger picture, when teams or entire organizations prioritize civility, it creates a safer work environment. Fewer unsafe actions occur, and there are even fewer injuries. Clark (2020) highlighted that when teams value civility, it helps reduce negative behaviors, even when faced with challenges.

### What are the stronger relationships between Workplace Civility and other work-related variables?

The meta-analytical review underscores the profound impact of Workplace Civility on the overall well-being and satisfaction of employees. A workplace climate rooted in respect and civility cultivates perceptions of improved job conditions. These positive perceptions are not just peripheral; they directly contribute to greater affective commitment to the organization and deter thoughts of leaving, as substantiated (Agarwal et al., 2023).

However, when delving into the association between Workplace Civility and health outcomes, we observed a nuanced pattern. Mental health indicators registered a notably high effect size, emphasizing the direct link between civility and psychological well-being. In stark contrast, physical health outcomes showcased minimal correlations with civility. This dichotomy resonates with Eriksen and Ursin’s (1999) findings, highlighting the differential impacts of subjective self-perceptions versus objective measurements. Liu (2020) proposed an intriguing perspective, suggesting that the relationship between health and civility might be reciprocal. Simply put, while a civil environment can promote health, healthier employees might also foster a more civil environment.

Yet, a significant lacuna in our understanding remains. While a plethora of research has dissected the negative aspects of organizational behavior, the interplay between civility and incivility remains enigmatic. Many studies have taken the simplistic route of labeling civility as the direct antithesis of incivility without substantial empirical backing. This presumption needs re-evaluation. A more rigorous exploration is essential to delineate the intricate dynamics of civility and incivility in professional settings.

### What strategies and interventions effectively promote and maintain Workplace Civility?

The present systematic review of workplace civility emphasizes several pivotal strategies that promote a civil work environment. Leadership stands central to driving civility. By embodying respect and setting clear civility benchmarks, leaders ensure a culture of mutual respect, augmented by open communication lines for employees’ concerns (der Kinderen et al., 2020). Effective communication, where employees engage actively and respectfully, sets the tone for mutual respect (Di Fabio and Kenny, 2016). While conflict is inherent to workplaces, its management is crucial. Skills acquired through conflict resolution training foster constructive discussions and harmonious resolutions (Gazica and Spector, 2016). A robust foundation is further established by clear workplace policies, which, when consistently enforced, ensure a professional and respectful culture (Palazzeschi et al., 2018).

Training sessions, especially around diversity and leadership, play a pivotal role in equipping employees to interact respectfully across varied backgrounds and in managerial capacities (Abd-Elrhaman and Ghoneimy, 2019; Gupta and Singh, 2021). Furthermore, celebrating employees’ positive behaviors through recognition programs fortifies a culture of appreciation and respect (Alamelu et al., 2017). Lastly, addressing workplace stress is vital. Comprehensive employee wellness initiatives that encompass physical and mental well-being mitigate stress and underscore the value placed on employees, fostering a supportive culture. In sum, a blend of strategies, spanning leadership commitment to employee wellness programs, can craft a civil workplace, driving a thriving organizational atmosphere.

### What gaps exist in the literature on Workplace Civility, and which areas require further research using the TCCM framework?

The present review highlighted key areas in the ongoing exploration of workplace civility through the lens of the TCCM framework. Theoretically, the CREW model has been dominant, casting civility as part of a broader scope (Osatuke et al., 2009; Savadkouhi et al., 2021; Sawada et al., 2021). However, this often overlooks the unique elements of civility on its own. There’s a pressing need to clarify how civility interacts with constructs like Positive Organizational Behavior due to shared characteristics.

In terms of study characteristics, a Western bias, primarily focusing on healthcare professionals, limits our understanding. The relatively small pool of studies, combined with diverse methodologies, hampers consistent conclusions. These challenges underscore the need for broader, more inclusive research. Contextually, the organizational environment plays a pivotal role in shaping civility. Workplaces that champion civility often foster satisfied, empowered employees (Andrade et al., 2017). Meanwhile, a culture of competitiveness might inadvertently boost uncivil behaviors. Methodologically, while empirical studies dominate, there’s a gap in understanding the deeper nuances of civility. Though some tools and interventions show promise, the field requires more structured and evidence-backed programs (Oppel and Mohr, 2020).

In conclusion, the growing interest in Workplace Civility is a call to refine our theoretical frameworks, broaden research horizons, and develop impactful interventions. Addressing these areas can lead to improved workplace well-being and organizational success.

### Limitations of the present review and meta-analyses

The present systematic review, though comprehensive in its approach, bears several limitations that merit consideration. First, the use of only the terms “civility” and “relation” might have restricted the range of articles retrieved, potentially omitting studies that explore workplace civility using different terminology or constructs. Second, even though we included studies written in English, Portuguese, French, Italian, or Spanish, it might have excluded potential valuable insights from studies written in other languages.

Third, the decision to exclude works on politics and law, economic costs, and students might limit the understanding of civility’s broader context. Moreover, by omitting studies focusing solely on incivility, the review might not capture the full spectrum of behaviors and their implications in the workplace. Fourth, while the study sources spanned multiple disciplines, there might be a geographical bias if a significant proportion of the studies are from particular regions, thereby not providing a global perspective on the concept. Fifth, the focus was mostly on formal publications like articles, doctoral theses, and book chapters. Some potentially valuable gray literature or less formal writings might have been overlooked.

Sixth, the registration protocol, though robust, does not guarantee uniform interpretation. Variability in coding, especially in the subjective areas, might introduce inconsistencies in the data extracted and subsequently analyzed, specifically due to the absence of a second participant researcher. In conclusion, while this review offers a comprehensive look into the concept of workplace civility, the limitations highlighted underscore the complexities of systematic reviews and the need for continual refinements in methodologies. Future research could consider expanding the search terms, languages, and sources to yield a richer, more diverse pool of studies on the topic.

## Conclusion

Our review found that there is less research on civility in the workplace compared to incivility in the workplace. Further research is needed to clarify its definition, establish a clear theoretical framework, and develop scales for measuring it. Additionally, exploring ways to promote civility at work can improve workers’ physical and mental wellbeing, reduce burnout and absenteeism (Leiter M. P. et al., 2011; Leiter et al., 2012), and save companies billions of dollars each year.

### Implications for future research, intervention, and social policymakers

The present research has important implications for intervention strategies aimed at promoting respectful behavior in the workplace. Our findings indicate that establishing and maintaining civility is a complex process involving personal, work-related, and social factors that interact with one another (Leiter et al., 2011). Therefore, interventions should not solely target individual workers but should also encompass groups and organizations. While certain personality traits or orientations may assist employees in managing workplace stress, our analysis suggests that an organizational culture that values civility is more effective in encouraging such behavior among employees. It is crucial, therefore, to assess organizational cultures and identify those that are more likely to tolerate impolite and aggressive conduct and intervene to promote civility (Liu et al., 2020). Intervention programs should be comprehensive and open to innovative methods, including mindful speech. Furthermore, our research on the importance of intervention is applicable to college students as well. Studies indicate that civility is a predictor of positive outcomes for high school and college students and can be enhanced through various programs.

Regarding the implications for policymakers, we recommend that future studies focus on examining gender disparities in Workplace Civility (Apaydin et al., 2022). Since civility can be perceived at both the individual and group levels, gender differences may emerge among individuals based on their gender and among organizations that differ in cultural dimensions such as masculinity versus femininity. Organizations that value masculinity may prioritize competitiveness over consensus and exhibit less cooperation and concern for the well-being of others (Clark and Walsh, 2016). On the other hand, organizations that value femininity may be more consensus-oriented, modest, and cooperative, promoting stronger cultural norms of civility in a less hierarchical, male-dominated, or competitive environment (Clark, 2014; Montalvo, 2014; Campbell et al., 2021). Additionally, there may be differences in civic behavior at work among employees from different national cultures due to variations in implicit norms for expressing disagreement between collectivistic and individualistic cultures (Costa, 2014; Liu, 2020; Ahmed Elsayed et al., 2021; Alam et al., 2021; Gupta and Singh, 2021; Hossny and Sabra, 2021; Sawada et al., 2021). In today’s diverse workplaces, understanding these differences can enhance communication and collaboration among workers (Chandolia and Anastasiou, 2020; Lv et al., 2020; Toscano et al., 2020). Finally, empirical research should investigate the potential impact of leaders’ responses when confronted with employees’ uncivil behavior (Porath et al., 2015; Laschinger and Read, 2016), as these reactions are observed by colleagues and may influence long-term outcomes, such as perceptions of organizational justice (Yanchus et al., 2017).

To sum up, the study emphasizes that promoting civility at work can lead to healthier organizations and happier workers (Di Fabio et al., 2016), while preventing loss of human capital.

## Author contributions

XP: Conceptualization, Data curation, Formal analysis, Funding acquisition, Investigation, Methodology, Project administration, Resources, Software, Supervision, Validation, Visualization, Writing – original draft, Writing – review & editing.
